# Case Report: The effect of asplenia on the response to influenza vaccination and passive transfer of immunity in an adult female pig

**DOI:** 10.3389/fimmu.2025.1568142

**Published:** 2025-04-28

**Authors:** Michael C. Rahe, John J. Byrne, Danielle Meritet, Erika Gruber, Stephanie N. Langel, Elisa Crisci

**Affiliations:** ^1^ Department of Population Health and Pathobiology, College of Veterinary Medicine, North Carolina State University, Raleigh, NC, United States; ^2^ Center for Global Health and Diseases, Department of Pathology, Case Western Reserve University, Cleveland, OH, United States

**Keywords:** asplenia, pig, IgM, antibody responses, influenza virus

## Abstract

Asplenia is an important cause of morbidity and mortality in humans. However, there are only very rare examples of this condition reported in domesticated species. Here we present a case of asplenia, diagnosed at necropsy, in a crossbred adult female pig from an influenza vaccine study. The humoral antibody response, including immune response to an influenza A virus vaccine, was characterized and compared to a parity-matched pig from the same study. The antibody profiles, lower total IgM with similar levels of IgG, were remarkably similar to those described in human patients with asplenia. However, in response to vaccination, the asplenic pig showed a robust hemagglutinin-specific IgM response with lower levels of IgG and IgA. These results were mirrored in the passively transferred antibody profiles of the asplenic dam’s piglets. This constitutes the first case of congenital asplenia described in the pig.

## 1 Introduction

The spleen is a large blood-filtering secondary lymphoid organ that contributes heavily to both the innate and adaptive immune response to infection (1). The splenic red pulp (rich in red blood cells) and white pulp (rich in lymphocytes) are categorized as separate anatomic structures but between which there is frequent interaction (2). Specifically, at the margins of the white pulp (marginal zone), there are heterogenous populations of IgM^+^ naïve and memory B cells that are uniquely positioned for responding to both systemic bacterial and viral infections (3, 4). These B cells help drive an IgM response to blood-borne infection that can be important for linking the innate and adaptive immune response through the opsonization of antigens (5).

Asplenia (absence of the spleen) is an important cause of morbidity and mortality in humans, as the lack of this blood-filtering organ predisposes patients to systemic infection with encapsulated bacteria (6). Most cases of asplenia are due to splenectomy following trauma-related injuries to the spleen (1). Rarely, asplenia can also be congenital and caused by several genetic disorders (6–8). There is currently no natural animal model of congenital asplenia. Only rare cases of suspected congenital asplenia have been reported in dogs and cattle in numbers too low to identify sex or breed predisposition (9, 10).

Here we report a case of asplenia in a mature crossbred female pig, a subject in an influenza vaccine research study, presenting with an altered humoral immune profile. At no point during the 70-day-long study did the pregnant and then lactating animal show any clinical signs that warranted treatment. The absence of spleen was only noted at gross necropsy. Follow-up testing characterized the effect that asplenia had on this animal’s general humoral immune profile, the response to influenza vaccination, and the transfer of immunity from dam to piglets with remarkable similarities and differences to the antibody profiles of previously reported human asplenic patients.

## 2 Case presentation

Two first-parity gilts were raised as part of a closed breeding herd at the North Carolina State University Swine Education Unit (SEU). The gilts were bred via artificial insemination at SEU, confirmed pregnant by ultrasound examination, and then transported to a BSL-2 laboratory animal facility at the NCSU College of Veterinary Medicine (CVM) where they were subjects in an influenza vaccine trial evaluating the transfer of maternal anti-influenza antibodies. Upon arrival at the CVM, the gilts were acclimated for 5 days in one pen with twice-daily feeding, free access to water, and monitoring of rectal temperature. The temperature levels were normal throughout the acclimation and immunizations, ranging from 37.8°C to 38.8°C, with minimal to no difference between the two animals.

**Figure 1:**
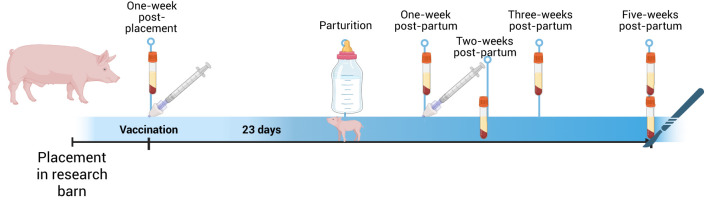
Experimental timeline of influenza vaccination and passive transfer study. Created in BioRender. Rahe, M. (2025). https://BioRender.com/q77z967.

At 1 week post-placement and approximately 3 weeks pre-partum, serum was collected, and both gilts were vaccinated intranasally (IN) with a monovalent proprietary influenza vaccine (dose: 0.95 × 10^11^ IU in 1 mL) (**Figure 1**) and monitored for vaccine-associated reaction with no difference between the two animals. At 20 days post-arrival, the gilts were placed in farrowing crates. At 9 days later, a split dose of 10 mg of Lutalyse (Dinoprost, Zoetis) was administered via intramuscular (IM) injection in the post-auricular neck muscles per the manufacturer’s recommendations (11). Both gilts farrowed on the following day. The asplenic gilt, hereafter identified as Asp, gave birth to 11 piglets. The normal gilt (Nor) gave birth to 16 live piglets, with two piglets undergoing humane euthanasia with an intravenous (IV) injection of barbiturate due to inability to stand and/or ambulate after birth. To decrease the number of piglets nursing from Nor, two piglets were cross-fostered from Nor to Asp shortly after birth. However, it is likely that both piglets suckled from Nor prior to being moved. Five pigs in the Asp’s litter, including the fostered ones, died within the first week of life due to various causes, mostly traumatic injuries.

Colostrum (15 mL) was collected from each dam during farrowing. At 1 week postpartum, the dams were given their second dose of the IN vaccine (dose: 0.5 × 10^11^ IU in 1 mL) and monitored for vaccine-associated reaction, with no difference between the two animals. Oxytocin was administered IM in the neck muscles each week at 1–5 weeks for milk collection. Blood was collected from dams at 1, 3, and 5 weeks postpartum and from piglets at 2 and 5 weeks of age. Five weeks after farrowing, the piglets were euthanized with an IV injection of barbiturate (pentobarbital sodium at 1 mL/4.5 kg). Three days later dams were sedated with an IV injection of ketamine (100 mg/ml) and xylazine (100 mg/ml) at 4 mg/kg and then euthanized with IV injection of barbiturate (pentobarbital sodium at 1ml/4.5kg).

The two dams presented for necropsy in good body condition with no to minimal autolysis. Nor weighed 159 kg and Asp weighed 200 kg. Asp was necropsied first, and the absence of a spleen was confirmed following an extensive gross examination of the abdominal cavity and the rest of the carcass. A single fibrous adhesion was noted between the cardia of the stomach and the intestinal mesentery that contained an approximately 4-cm-diameter, round, tan soft mass (presumed mesenteric lipoma). On the cut surface, the center of the mass was dark brown and firm (necrosis with mineralization). Several small, approximately 0.5–1.0 cm in diameter, dark red to purple nodules (suspected hemal nodes) were noted within the greater omentum near its attachment to the stomach and collected for single-cell suspensions. The remainder of the necropsy was unremarkable, and a collection of fresh and fixed tissues (mammary gland, nasal mucosa, lung, trachea, hemal nodes, submandibular lymph node, and ileum) was performed for further evaluation. Nor was necropsied second with the collection of fresh and fixed tissues (mammary gland, nasal mucosa, lung, spleen, and submandibular lymph node) for further evaluation. No gross lesions or abnormalities were noted during the necropsy.

To assess the effect of asplenia and vaccination on humoral immunity, total antibody isotype levels (IgM, IgA, and IgG) and influenza hemagglutinin (HA)-specific antibodies were evaluated by comparative ELISA. The transfer of influenza HA-specific maternal antibodies to piglets was also assessed. U-bottom 96-well Microtiter™ Microplates (Thermo scientific) were coated with 2 µg/well of purified capture IgG, IgA, IgM (Bio-Rad) or HA-trimer (eENZYME) where applicable in 1× KPL Coating Solution (VWR) and incubated at room temperature for 2 h. The plates were washed three times with 0.1% PBST after each incubation. The protein-coated plates were blocked with 100 µL blocking buffer (1% fish gel in 0.1% PBST) and incubated at room temperature for 1 h. To evaluate total immunoglobulins, serum and colostrum were diluted 1:100 and 1:1,000, respectively, in blocking buffer, added to the plate, and incubated at room temperature for 1 h. When evaluating HA-specific IgG and IgA, isolated IgG and IgA from the respective sample types were diluted at the concentration of 5 µg/100 µL in blocking buffer (isolation protocol at dx.doi.org/10.17504/protocols.io.yxmvmee6ng3p/v1) (12). Evaluation of HA-specific IgM required serum and colostrum dilutions at 1:100 and 1:400, respectively, in blocking buffer. HA-IgM antibody binding was detected by adding 100 µL of in-house biotin-conjugated purified-HA-trimer diluted 1∶100 in blocking buffer at room temperature for 1 h, followed by a separate incubation with 1:100 diluted streptavidin-HRP for 30 min. All other antibody binding were detected by adding 100 µL of anti-porcine IgG-, IgA-, or IgM-specific HRP-conjugated antibodies (Bio-Rad) diluted 1∶4,000 in blocking buffer at room temperature for 1 h. The colorimetric detection was evaluated using 100 µL of KPL SureBlue Reserve™ TMB Microwell Substrate (VWR) per well. The analysis was performed on either the end of exposure (30-min incubation) requiring optical density (OD) evaluation utilizing the Biotek Microplate spectrophotometer set at 650 nm or when the reaction was stopped by the addition of 100 µL of KPL SureBlue Reserve™ STOP (VWR), and the OD was measured at 450 nm.

**Figure 2:**
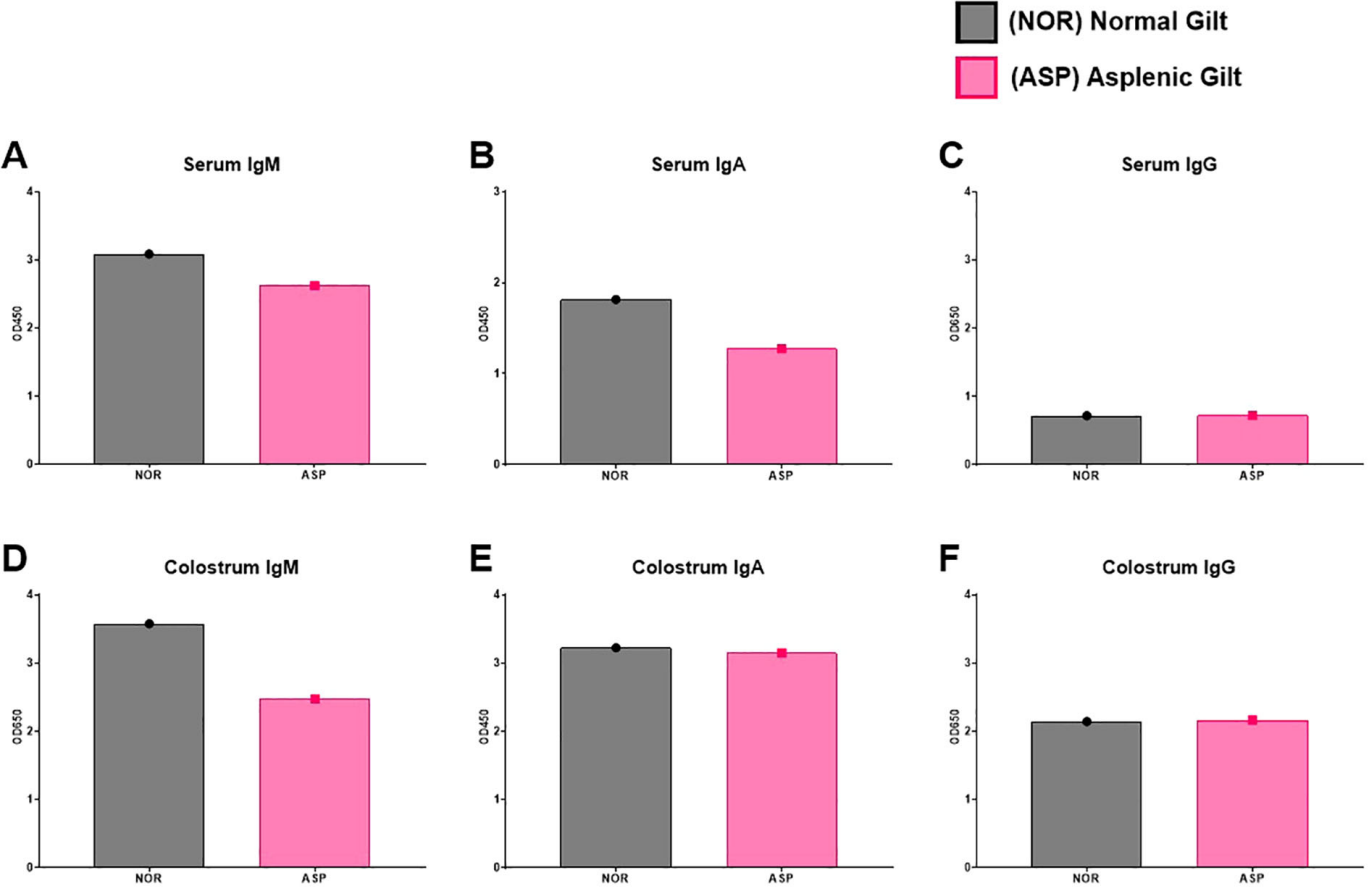
Total antibody responses. **(A)** ELISA results of Nor and Asp serum IgM level 2 weeks post-boost (3 weeks post-parturition). **(B)** ELISA results of Nor and Asp serum IgA level 2 weeks post-boost (3 weeks post-parturition). **(C)** ELISA results of Nor and Asp serum IgG level 2 weeks post-boost (3 weeks post-parturition). **(D)** ELISA results of Nor and Asp colostrum-derived IgM level. **(E)** ELISA results of Nor and Asp colostrum-derived IgA level. **(F)** ELISA results of Nor and Asp colostrum-derived IgG level. Optical density (OD) 450 and 650 nm. Nor, *N* = 1. Asp, *N* = 1. No statistics were performed on the N-of-1 evaluation.

**Figure 3:**
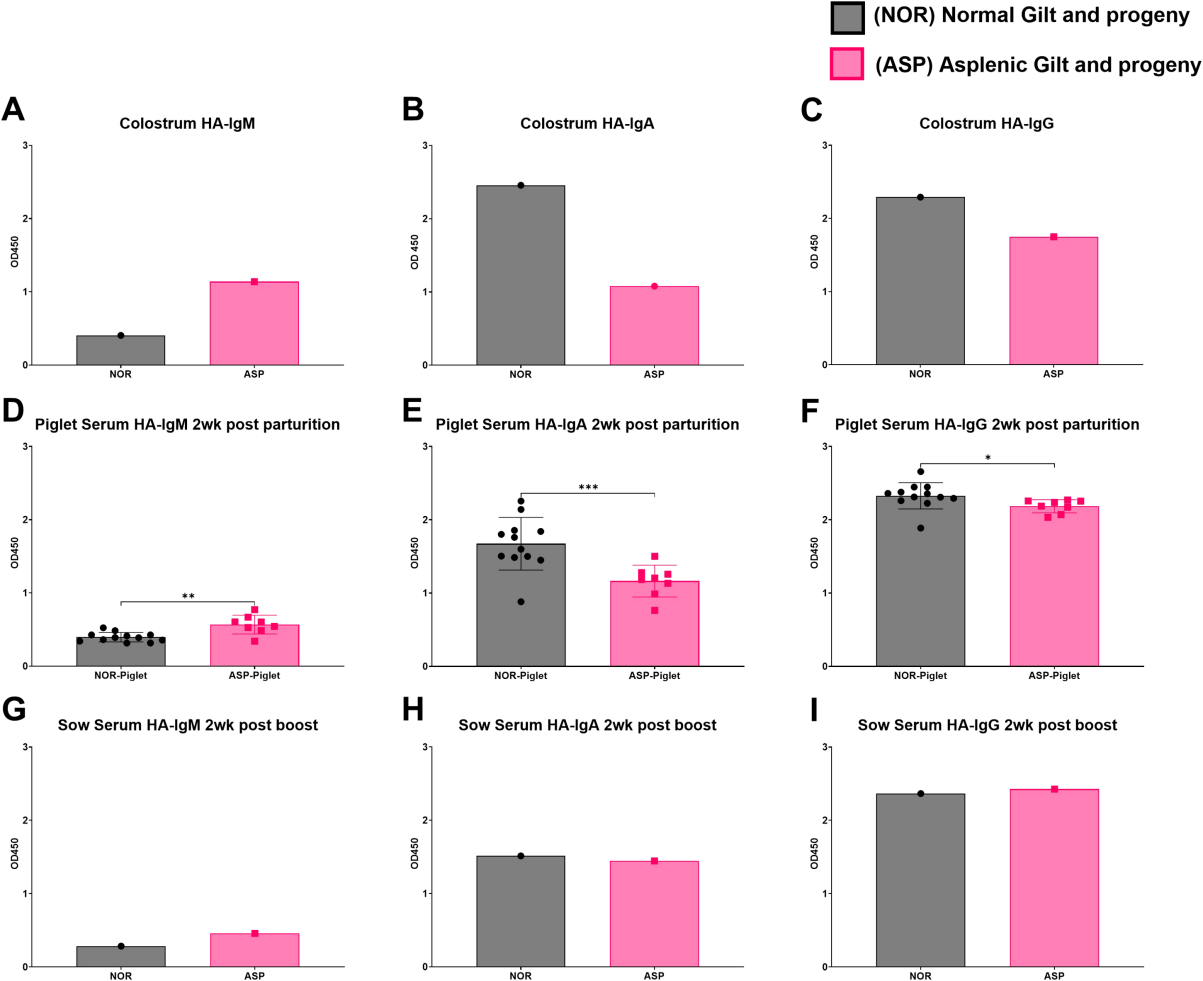
Hemagglutinin (HA)-specific antibody responses. **(A)** ELISA results of Nor and Asp colostrum-derived influenza HA-specific IgM; colostrum dilution 1:400. **(B)** ELISA results of Nor and Asp colostrum-derived influenza HA-specific IgA. Antibodies were isolated and used at a concentration of 5 µg/100 µL. **(C)** ELISA results of Nor and Asp colostrum-derived influenza HA-specific IgG. Antibodies were isolated and used at a concentration of 5 µg/100 µL. **(D)** ELISA results of Nor and Asp respective piglet serum (1:100)-derived influenza HA-specific IgM 2 weeks post-birth. **(E)** ELISA results of Nor and Asp respective piglet serum (1:100)-derived influenza HA-specific IgA 2 weeks post-birth. **(F)** ELISA results of Nor and Asp respective piglet serum (1:100)-derived influenza HA-specific IgG 2 weeks post-birth. **(G)** ELISA results of Nor and Asp serum derived influenza HA-specific IgM 2 weeks post-boost (3 weeks post-parturition); serum dilution 1:100. **(H)** ELISA results of Nor and Asp serum-derived influenza HA-specific IgA 2 weeks post-boost (3 weeks post-parturition). Antibodies were isolated and used at a concentration of 5 µg/100 µL. **(I)** ELISA results of Nor and Asp serum-derived influenza HA-specific IgG 2 weeks post-boost (3 weeks post-parturition). Antibodies were isolated and used at a concentration of 5 µg/100 µL. Optical density (OD), 450 nm. Nor, *N* = 1. Asp, *N* = 1. NOR-piglet mean ± SD. *N* = 12. ASP-piglet mean ± SD. *N* = 8. Statistics: Welch’s *t*-test performed using GraphPad Prism version 10.4.1 for Windows, GraphPad Software, Boston, MA, USA. www.graphpad.com. *p < 0.05; **p < 0.01; ***p < 0.001.

In both serum and colostrum, Asp had lower levels of total IgM with similar amounts of total IgG (**Figure 2**). Asp had lower serum IgA but similar colostral levels to Nor (**Figures 2B, E**). The dams were vaccinated against influenza at approximately 3 weeks pre-partum and then boosted 1 week post-partum. Surprisingly, the colostrum collected from Asp had more HA-specific IgM compared to Nor (**Figure 3A**). This translated to Asp’s piglets having significantly more HA-specific IgM in serum at 2 weeks post-partum compared to Nor’s piglets (**Figure 3D**). The results for HA-specific IgA were inverse those of IgM, with Nor having more IgA than Asp in colostrum and piglets with significantly more HA-specific IgA in serum (**Figures 3B, E**). Nor also had somewhat higher HA-specific IgG levels in the colostrum, reflected in the significant difference in piglet serum levels of HA-specific IgG between litters (**Figures 3C, F**). At 3 weeks post-partum and 2 weeks after booster vaccination, evaluation of antibodies showed that Asp had slightly more serum HA-IgM than Nor with similar levels of both IgG and IgA (**Figures 3G–I**).

The serum chemistries collected both pre- and post-partum as well as at necropsy were unremarkable in both animals (**Supplementary Table S1**). Flow cytometry was performed on single-cell suspensions of the suspected hemal nodes to evaluate if they contained ectopic splenic tissue. The frequency of identified cellular populations was similar to those of the submandibular lymph node collected from the same animal with numerous CD21^+^ B cells, fewer CD3^+^ T, and a low population of CD163^+^ macrophages and different from the spleen frequencies observed from the Nor pig (**Supplementary Figure S1**). Further evaluation of Wright–Giemsa-stained cytocentrifuged cellular preparations from the hemal nodes of Asp and lymph node from Asp and an additional control animal revealed no discernible differences in cellular populations (**Supplementary Figure S2**).

Howell–Jolly bodies in red blood cells have previously been reported in humans with asplenia, as the loss of the spleen prevents the removal of these cells (13, 14). As blood smears were not available at this stage of investigation, Giemsa stains were performed on fixed sections of lung for both Asp and Nor to attempt to identify these very small nuclear remnants in the red blood cells of Asp. No definitive Howell–Jolly bodies were noted in the red blood cells in the evaluated sections.

## 3 Discussion

The pig spleen is similar to the human spleen in size, location in the abdomen, and regenerative capacity (15–18). The human and pig immune systems are also remarkably similar (19, 20). Therefore, it was not surprising to find similarities between human and porcine asplenic immunity in this case. Specifically, Asp and Nor had similar levels of total IgG, but markedly different IgM levels. The total antibody levels are very comparable to what have previously been shown in populations of vaccinated asplenic human patients versus controls (21). While the reduced serum total IgM in Asp could be attributed to the loss of the robust IgM^+^ B cell response found in the spleen, the comparatively higher colostral HA-IgM response to vaccination is puzzling. Perhaps the adaptive immune response to initial vaccination in the Asp pig was delayed compared to Nor, resulting in an IgM response that had not contracted yet. Additionally, the colostral HA-specific IgA and IgG levels in Asp are lower than those of Nor. These antibody levels were reflected in the maternally derived antibodies in each gilt’s respective piglets. The reason for the much lower HA-specific class-switched antibody response in Asp is unclear. A link between the spleen and the IgA response via a possible gut–spleen axis has previously been suggested (22, 23). Therefore, the absence of IgM^+^ splenic B cells may have limited the IgA response to IN vaccination. Interestingly, the noted large differences between the HA-specific antibody response between Asp and Nor were largely diminished 2 weeks after booster vaccination. However, given the limited sample size, the possibility that the observed results are influenced by natural variation in outbred animals cannot be excluded.

In the pig, the maternal transfer of antibodies from dam to piglets occurs via colostrum as the epitheliochorial placentation of the pig prevents transplacental antibody transfer. As expected, the serum levels of HA-specific antibodies in piglets mirror the colostrum levels of these antibodies, and asplenia appeared to only limit HA-specific IgA responses in colostrum. While maternal immunity against influenza appears to confer limited protection to piglets, the transfer of antibodies against encapsulated bacteria that cause systemic infections in pigs, such as *Streptococcus suis*, could be of concern in an asplenic animal (24). However, none of Asp’s piglets showed signs of systemic bacterial infection.

The majority of people with asplenia have undergone splenectomy. Congenital asplenia is a less common cause of asplenia and has been associated with several disorders (6). The cause of the asplenia in this pig was unclear and perhaps complicated by the discovery of the presumed lipoma in the anatomic location that would normally contain the spleen. Mesenteric lipomas have been described in humans and pigs, and while not invasive, they may invade or compress adjacent organs, such as the small intestine (25, 26). However, one affecting the spleen has not previously been reported. Lipomatous neoplasms of the spleen are rare in humans and have never been described in the pig (27). Additionally, based on the size of the mass and benign behavior, it would be highly unlikely that the lipoma effaced the spleen. Horses develop mesenteric lipomas that can twist around the intestines, resulting in infarction. While it may be possible for a mesenteric lipoma to cause strangulation of the vessels of the spleen resulting in infarction, this would have likely resulted in acute shock and subsequent death of the gilt rather than loss of the spleen and complete recovery (28).While splenectomy surgeries have been described and are performed by human surgeons on pigs to study various aspects of splenic trauma, blood loss, and reperfusion injury, this pig was not a subject in any such study, confirmed at necropsy by the lack of a surgical scar or evidence of prior surgery (29, 30). Additional possibilities for the apparent lack of a spleen could be splenic hypoplasia or an intrapancreatic accessory spleen. Splenic hypoplasia occurs most commonly in domestic animals as part of severe combined immunodeficiency (31). Intrapancreatic accessory spleen (IPAS) has been described in the context of wild boars infected with African swine fever virus (ASFV) (32). The data presented show that Asp was immunocompetent, and although we could not detect a visible alteration of the pancreas, a compensatory IPAS cannot be ruled out.

The suspected hemal lymph nodes were an unexpected finding and initially thought to be associated with asplenia of the gilt, as the formation of hemal nodes post-splenectomy has been previously described in the dog (33). The nodes may serve a compensatory function in blood filtering or erythrophagocytosis. While these roles cannot be ruled in or out in the asplenic gilt, hemal nodes have been reported before in normal pigs (34). Further histopathological investigation of the hemal nodes or the presence of Howell–Jolly bodies or nucleated red blood cells was not performed at the time of necropsy, as the focus of the study was evaluating the immune response to vaccination. The subsequent creation of single-cell suspensions of mononuclear cells from immune tissues required the removal of red blood cells. Therefore, there were no red blood cells in the described cellular smears of isolated cells.

The presented case demonstrates that asplenia occurs in pigs. Furthermore, this case appears to be congenital, suggesting the future possibility of an animal model for the study of asplenia. Considering the likely very low prevalence of this condition, the antemortem diagnosis of additional cases in the pig would be challenging. However, the identification of asplenic pigs at slaughter with the subsequent collection of material for genetic analysis may identify genes that could be bred or edited in the creation of an asplenic animal model.

## Data Availability

The raw data supporting the conclusions of this article will be made available by the authors, without undue reservation.

## Supplementary material

Supplementary Figure 1 Flow cytometry staining and gating strategy of Asp and Nor frozen tissues. Gating hierarchy from forward/side scatter (FSC/SSC) gate on total cells, the exclusion of doublets and dead cells to focus on CD3+ T cells, CD21+ B cells, and CD163+ macrophages. Comparison between unstained control hemal node with stained Asp hemal node, Nor lymph node, and Nor spleen. Staining with directly conjugated antibodies: mouse IgG1 anti-pig CD3 (PPT3) FITC (Southern Biotech), mouse IgG1 anti-pig CD21 (B-ly4) BV421 (BD Biosciences), mouse IgG1 anti-pig CD163 (2A10/11) PE (ThermoFisher), live/dead near-IR (APC-Cy7) (Invitrogen).

Supplementary Figure 2 Cytospin images. ×200. There were no discernible differences in the identified populations. No red blood cells were present due to the mononuclear cell isolation performed on these samples.
