# Supplementary figures and images for: Case Report: The effect of asplenia on the response to influenza vaccination and passive transfer of immunity in an adult female pig
Source: Front Immunol. 2025 Apr 28;16:1568142. doi: 10.3389/fimmu.2025.1568142 (PMC12066655; doi:10.3389/fimmu.2025.1568142)

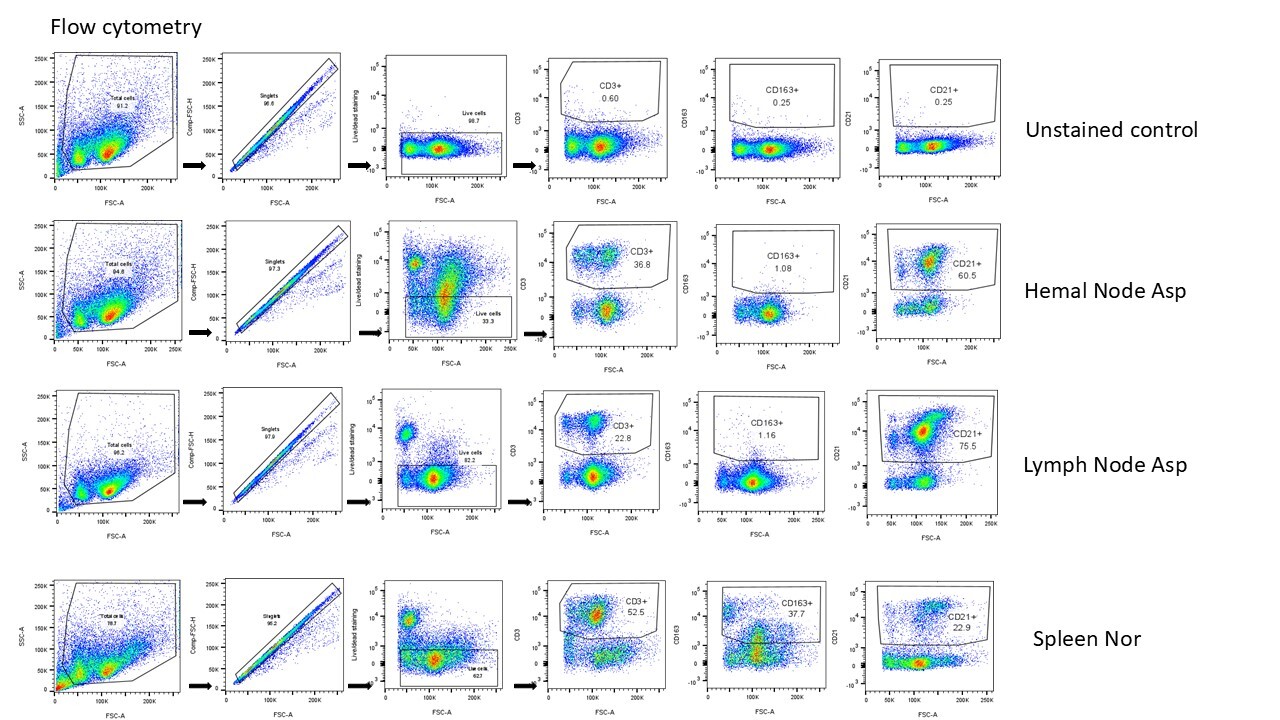

Supplement: Supplementary Figure 1 — Flow cytometry staining and gating strategy of Asp and Nor frozen tissues. Gating hierarchy from forward/side scatter (FSC/SSC) gate on total cells, the exclusion of doublets and dead cells to focus on CD3+ T cells, CD21+ B cells, and CD163+ macrophages. Comparison between unstained control hemal node with stained Asp hemal node, Nor lymph node, and Nor spleen. Staining with directly conjugated antibodies: mouse IgG1 anti-pig CD3 (PPT3) FITC (Southern Biotech), mouse IgG1 anti-pig CD21 (B-ly4) BV421 (BD Biosciences), mouse IgG1 anti-pig CD163 (2A10/11) PE (ThermoFisher), live/dead near-IR (APC-Cy7) (Invitrogen). [file Image1.jpeg]

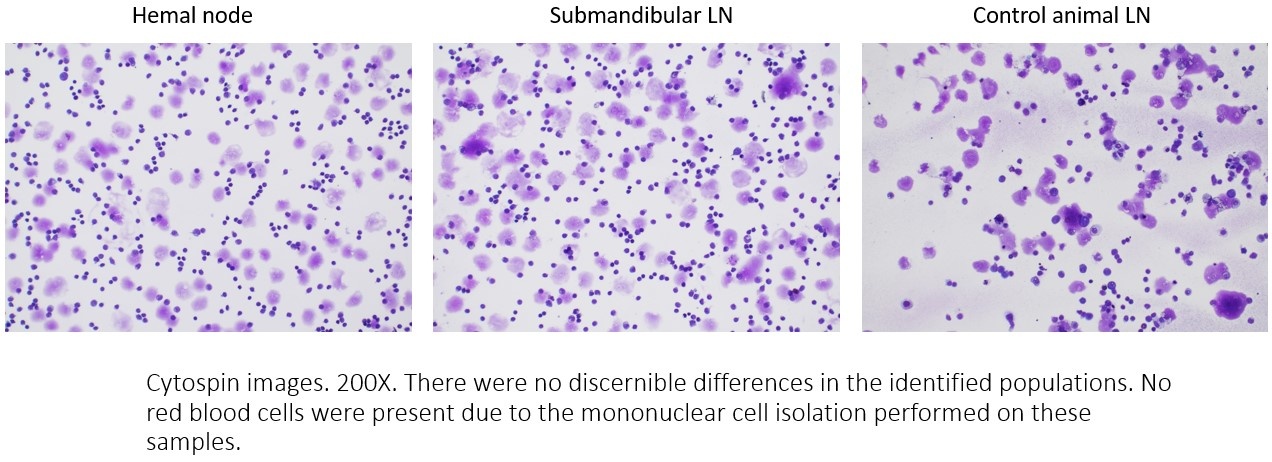

Supplement: Supplementary Figure 2 — Cytospin images. ×200. There were no discernible differences in the identified populations. No red blood cells were present due to the mononuclear cell isolation performed on these samples. [file Image2.jpeg]
